# Supplementary material for: A realistic mixture of ubiquitous persistent organic pollutants affects bone and cartilage development in zebrafish by interaction with nuclear receptor signaling
Source: PLoS One. 2024 Mar 28;19(3):e0298956. doi: 10.1371/journal.pone.0298956 (PMC10977810; doi:10.1371/journal.pone.0298956)
Supplement: S2 Table — Average in percentage and standard deviation values in percentage of the prevalence of micrognathia in zebrafish larvae at 5dpf. Mixed-Effect model (Treatment and Treatment*Phenotype), uncorrected Fisher’s LSD test. p values: * <0.05; ** < 0.01; *** <0.001, **** < 0.0001. Asterisk (*) when differences were found against Control, pound sign (#) when differences were found against PFAA alone. (DOCX) [file pone.0298956.s002.docx]

|  | Control | POP125× | PFAA | Br | Cl | PFAA+Br | PFAA+Cl | Br+Cl |
| --- | --- | --- | --- | --- | --- | --- | --- | --- |
| Severe | 0 | 30±3* | 11±11 | 0 | 0 | 5±5 | 15±15 | 0 |
| Mild | 15±5 | 49±15** | 42±2* | 26±4 | 43±17* | 60±2***^#^ | 75±5****^##^ | 35±5 |
| Normal | 85±5 | 21±12****^#^ | 47±13** | 74±4^#^ | 58±17* | 35±5**** | 10±10****^#^ | 65±5 |
