# Supplementary material for: A realistic mixture of ubiquitous persistent organic pollutants affects bone and cartilage development in zebrafish by interaction with nuclear receptor signaling
Source: PLoS One. 2024 Mar 28;19(3):e0298956. doi: 10.1371/journal.pone.0298956 (PMC10977810; doi:10.1371/journal.pone.0298956)
Supplement: Attachment — Submitted filename: Rebuttal letter to PLOSOne.docx [file pone.0298956.s005.docx]

PONE-D-23-36871

Response to Reviewers

A realistic mixture of ubiquitous persistent organic pollutants affects bone and cartilage development in zebrafish by interaction with nuclear receptor signaling.

Academic Editor

Additional information regarding key experimental details is needed, as pointed out by Reviewer 2. I also recommend that you consider the suggestion by Reviewer 1 to include a mechanistic diagram to explain how chronic POP exposure compromises bone mineralization.

Journal requirements:

1. When submitting your revision, we need you to address these additional requirements.

Please ensure that your manuscript meets PLOS ONE's style requirements, including those for file naming. The PLOS ONE style templates can be found at 
https://journals.plos.org/plosone/s/file?id=wjVg/PLOSOne_formatting_sample_main_body.pdf and 
<https://journals.plos.org/plosone/s/file?id=ba62/PLOSOne_formatting_sample_title_authors_affiliations.pdf>.

checked

2. We note that the grant information you provided in the ‘Funding Information’ and ‘Financial Disclosure’ sections do not match.

When you resubmit, please ensure that you provide the correct grant numbers for the awards you received for your study in the ‘Funding Information’ section.

3. Thank you for stating the following financial disclosure: 
 [This research was funded by the European Union’s Horizon 2020 research and innovation program under the Marie Skłodowska-Curie Innovative Training Network (ITN) program PROTECTED [Grant agreement No. 722634]. G.G-L. was a PROTECTED fellow, M.M. is a "Maître de Recherche" at "Fonds National de Recherche Scientifique (FNRS).].  
Please state what role the funders took in the study.  If the funders had no role, please state: ""The funders had no role in study design, data collection and analysis, decision to publish, or preparation of the manuscript.""

If this statement is not correct you must amend it as needed. 
Please include this amended Role of Funder statement in your cover letter; we will change the online submission form on your behalf.

done

4. Thank you for stating the following in the Acknowledgments Section of your manuscript: 
[This research was funded by the European Union’s Horizon 2020 research and innovation program under the Marie Skłodowska-Curie Innovative Training Network (ITN) program PROTECTED [Grant agreement No. 722634]. G.G-L. was a PROTECTED fellow, M.M. is a "Maître de Recherche" at "Fonds National de Recherche Scientifique (FNRS). The authors would like to thank the GIGA zebrafish platform (H. Pendeville-Samain) for taking care of and delivering the zebrafish larvae, the GIGA imaging platform for their help and support with microscopy, the GIGA genomic platform for sequencing, and the GIGA bioinformatics platform for data analysis.]
We note that you have provided funding information that is not currently declared in your Funding Statement. However, funding information should not appear in the Acknowledgments section or other areas of your manuscript. We will only publish funding information present in the Funding Statement section of the online submission form

Please remove any funding-related text from the manuscript and let us know how you would like to update your Funding Statement. Currently, your Funding Statement reads as follows: 
[This research was funded by the European Union’s Horizon 2020 research and innovation program under the Marie Skłodowska-Curie Innovative Training Network (ITN) program PROTECTED [Grant agreement No. 722634]. G.G-L. was a PROTECTED fellow, M.M. is a "Maître de Recherche" at "Fonds National de Recherche Scientifique (FNRS).].

Please include your amended statements within your cover letter; we will change the online submission form on your behalf.

5. We note that Figure(s) 1, 2, 3 and 4 in your submission contain copyrighted images. All PLOS content is published under the Creative Commons Attribution License (CC BY 4.0), which means that the manuscript, images, and Supporting Information files will be freely available online, and any third party is permitted to access, download, copy, distribute, and use these materials in any way, even commercially, with proper attribution. For more information, see our copyright guidelines: http://journals.plos.org/plosone/s/licenses-and-copyright.

We would like to emphasize that all the figures presented in this manuscript are originals, that have never been published elsewhere. We actually were in doubt concerning the schematic drawings of alcian blue stained zebrafish larvae in Fig. 1, so we replaced those with a home-made scheme. Please do check that again.

Reviewers' comments:

Reviewer 1

We would like to thank the reviewer for his useful comments and corrections. It really helped us to improve the manuscript.

**Comments to the Author**

Review Comments to the Authors

Reviewer #1: In this research article, Muller and colleagues elaborated the impact of a mixture of ubiquitous persistent organic pollutants (POPs) on bone and cartilage development in zebrafish. This is a complex and specific area of study that involves understanding the effects of environmental contaminants on biological systems. This type of research is crucial for understanding the potential risks and mechanisms of environmental contaminants on developmental processes in organisms, including potential implications for human health. Hence, this research article is relevant for the field.

However, the more informative approaches could be performed such as assessing the expressing of bone related genes (Sox9a, SPP1/OPN, and Col1a1) in addition to the RNA seq (https://doi.org/10.1016/J.SCITOTENV.2021.147989). The authors could compare the results of the reference and highlight their results in Discussion. Besides, did POP induce any oxidative stress, which could be assayed using oxidative stress markers like acridine organe staining, and GSH, SOD, etc metabolic assays. These analysis need to be done to understand the role of POPs in influencing the biological performance of zebrafish.

We appreciate this comment. Note that most of the time, antibodies for specific proteins are not available in zebrafish. We did however perform RNA-Seq transcriptome analysis, and we now have reanalyzed these results in the light of the Reviewer's comment. This is now added in the discussion " A decrease in mineralized bone formation, as observed here, may be due to inhibition of osteoblast proliferation, differentiation, inhibition of ECM deposition, or the final step of mineralization (Fig 8) [36, 82, 83]. Here, the transgenic line *Tg(col10a1a:col10a1a-GFP)* was used to reveal total (i. e. unmineralized and mineralized) bone ECM, combined with live staining of the mineralized ECM [38]. This experiment revealed that the POP125× more strongly affected deposition of the unmineralized ECM than the subsequent mineralization. This is consistent with the observed decrease in expression of the collagen and collagen maturation enzyme genes. Furthermore, manually checking the RNA-Seq results from treated larvae [22] did not reveal any changes in expression of genes involved in osteoblast differentiation, such as *sox9a, runx2b, sp7,* or *spp1* [84]. Similarly, genes involved in inflammation or oxidative stress (*sod, gsh*) were not significantly affected. In conclusion, our results strongly suggest that mainly the deposition of collagenous bone ECM is affected by the POP125× mixture tested here in 5 dpf zebrafish larvae (Fig 8)." (lines 440-453). We also added a schematic view of these conclusions (Fig 8). In addition, we now refer to the cited, interesting paper in lines 408-409.

The authors state in results “Bone mineralization is compromised following 4-day chronic exposure.” This need to be justified with proper clarification citing other references. What lead to such bone demineralization? A mechanistic diagram would be better to explain.

These were our observations, which we now interpret more clearly in the discussion. The formulation was indeed misleading. We replaced this title with "Mineralized bone formation is compromised ….", (line 181) as at this stage it may be premature to focus on one of the steps in mineralized bone formation: ECM deposition or mineralization. We now also added a diagram to better explain our final conclusion that mainly ECM deposition is affected (Fig 8 and see above).

Other Comments

Line 65: Sentence needs to be modified

Done (line 65)

Line 68: “have addressed” instead of “are addressing”. “have researched” instead of researching. Sentence needs to be modified.

Done (lines 69-71)

Line 71: “such as this particular” instead of “such as that this particular”

Sentence changed (line 72-75).

Line 73: “receptor [19], and enhances” instead of “receptor [19], enhances”

Done (line 80)

Line 81- 86: Segregate the sentence starting from “Some POPs” and ending with “for instance” into two different sentences.

Done (lines 83-88)

Line 136: “different sub-mixes were tested” instead of “we tested the different sub-mixes”

Done line(150)

Line 149: “observed” instead of “photographed”

Done (line 163)

Line 152: Use technical terms instead of “normal”.

Replaced by "Unaffected" (lines 166-167)

Line 169-171: Sentence starting with “After the different” and ending with “ staining protocol” needs to be modified.

Done (lines 184-186)

Line 172: “The area of the opercle was measured” instead of “We measured the area”

Done (line 188)

Line 173 - 175: Sentence starting with “Surviving individuals” and ending with “ including controls” needs to be modified.

Done (line 189-190)

Line 187-188: “the transgenic line Tg were used” instead of “we decided to use”

Done (line 204)

Line 192-193: Sentence starting with “In addition” and ending with “bone structures” needs to be modified.

Done (line 208-210)

Line 193: Use technical terms instead of “Looking at”

Done (line 210)

Line 195: Use technical terms instead of “Surprisingly, looking at”

Done (line 211-212)

Line 196 -197: Sentence needs to be modified

Done (line 211-214)

Line 198-199: Sentence starting with “Calculating” and ending with “opercle area” needs to be modified and use “The obtained ratio of” instead of “Calculating the ratio between”

Done (line 214-216)

Line 199: “Further, this analysis indicates” instead of “Taken together”. “cause” instead of “is causing”

Done (line 215)

Line 220: “the list of DEGs were reanalysed” instead of “we reanalysed the list of DEGs”

Done (line 236)

Line 223: “only GSEA identified” instead of “only GSEA also identified”

Done (line 239)

Line 224 - 225: Sentence starting with “In addition” and ending with “carried out” needs to be modified

Done (line 240-241)

Line 227: Rephrase “caught our attention”

Done (line 243)

Line 232-233: “a network of these zebrafish genes were constructed” instead of “we constructed a network of these zebrafish genes”

Done (line 248-249)

Line 236: “It was observed that” instead of “It clearly appears”

Done (line 252)

Line 253: Rephrase “attracted our attention”

Done (line 269)

Line 259-260: Rephrase the sentence starting with “Strikingly” and ending with “treatment”

Done (line 275-276)

Line 260: Strike out the term “Obviously”

Done (line 297)

Line 281 - 288: Strike out the term “we” and rephrase the sentences.

Done (line 297-208)

Line 307: Strike out the term “our” and specify the list name

Done (line 323)

Line 314: “focuses” instead of “will focus”

Done (line 331)

Line 318, 320, 322, & 324: Strike out the term “we” and rephrase the sentences

Done (line 334-340)

Line 328: Rephrase “To the best of our knowledge”

Done (line 344-345)

Line 329 & 331: Strike out the term “we” and rephrase the sentences

Done (line 345-348)

Line 364: “In the experiments carried out mild to severe effects were observed on” instead of “In our experiments, we observed”

Done (line 381)

Line 372: Strike out the term “looking” and rephrase the sentence

Done (line 389)

Line 374 -375: Strike out the term “we” and rephrase the sentences

Done (line 391-392)

Line 392: Rephrase “We previously showed”

Done (line 393)

Line 395-396: Strike out the term “we” and rephrase the sentences

Done (line 414)

Line 405 & 419: Strike out the term “our” and rephrase the sentence

Done (line 423 and 437)

Line 423: Rephrase “our toolbox”

Deleted as it was repetition (line 440)

Line 428, 430, & 481: Strike out the term “we” and rephrase the sentences

Done or deleted as repetition (line 440-453)

Line 489-490: Rephrase “no less than”

Done (line 535)

Line 502: Strike out the term “we” and rephrase the sentences

Done (line 547)

Some relevant works that can be included and commented in the manuscript are:
Verma, S.K., Nisha, K., Panda, P.K., Patel, P., Kumari, P., Mallick, M.A., Sarkar, B., Das, B., 2020. Green synthesized MgO nanoparticles infer biocompatibility by reducing in vivo molecular nanotoxicity in embryonic zebrafish through arginine interaction elicited apoptosis. Sci. Total Environ. 713, 136521. https://doi.org/10.1016/J.SCITOTENV.2020.136521
Makkar, H., Verma, S.K., Panda, P.K., Jha, E., Das, B., Mukherjee, K., Suar, M., 2018. In Vivo Molecular Toxicity Profile of Dental Bioceramics in Embryonic Zebrafish (Danio rerio). Chem. Res. Toxicol. 31, 914–923.

<https://doi.org/10.1021/ACS.CHEMRESTOX.8B00129/SUPPL_FILE/TX8B00129_SI_001.ZIP>

We have read these two remarkable works, however, as explained above and now in the discussion (lines 430-453), it seems as if the effects described here do not relate to those involved in these effects. Makkar et al clearly focus on the estrogen pathway, while Verma et al focus on oxidative stress and apoptosis. As we now explain, neither of these was detected in our RNA-Seq analysis.

Reviewer #2: The manuscript by Guerrero-Limon et al describes the toxicity testing of a mixture of 29 POPs at environmentally relevant concentration in zebrafish, with a focus on the bone and cartilage development. The manuscript is well written and the studies are logical and appropriate. There are some comments/clarification needed throughout:

Abstract:
- The first sentence does not add any value

Changed (line 33-34)

- The number of POPs in the mixture should be stated

Done (line 37)

Introduction:
- There is not enough context to explain why the effects on bone and cartilage is of concern for POPs. LN 79-81 talks about how there isn’t any studies. So why should we care?

Added " Indeed, their diverse effects on general metabolism (e.g. as endocrine disruptors) carry the risk of affecting skeletal development (scoliosis, craniofacial) as well as pathologies such as osteoporosis or osteoarthritis [23]" in line 90-94

- Some more context on this “realistic mixture” is needed

Done (line 72-78)

Methods:
- LN 467: a statement of how the experiment is designed is referenced, but there needs to be more detail of how the solutions were made (LN 461), and experimental chambers, etc. There is barely enough detail to understand there are 7 mixtures created and tested throughout. What concentration of DMSO was used? A blanket statement of “treated for at least 96h” is insufficient. This does not get anywhere close to having enough detail for another lab to reproduce.

We now give more details about the experimental details (line 504-515)

- LN 475 – what concentration of MS-222?

Done (line 519)

Results:
- There needs to be more information on what the 7 mixtures are, rather than just jumping in and saying “POP125+”. At the very least, figure 1 legend should be more descriptive of what the 7 mixtures/submixtures are.

We reformulated this part "Previous experiments have tested the total mixture of 29 POPs on zebrafish larvae [17], where a lethal concentration of LC50 = 386-fold the mean human blood concentration (POP386×) was found [22], while exposure to POP75× or POP125× resulted in more than 95% survival at 4 dpf. On the other hand, POP125× did affect behavior, heart rate, and eye development [22] and can be considered as a realistic scenario in highly exposed populations (e.g. sea food, environmental/ natural disaster). Thus, the POP125× mix (S1 Table) was chosen to expose WT zebrafish larvae during the first 4 days for studying the effects on skeletal development." in lines 119-125 and in line 150

- LN 167 – is 4 day exposure really considered “chronic” if staining happens at 10 dpf. I would state that as continuous exposure. Why the 10 dpf timepoint for bone mineralization?

Agreed. We changed it to continuous and explain why we looked at bone staining at 10 dpf. (line 182-186)

Discussion:
- This is a well written discussion. The only point that I would have liked to relate why finding structural chemical similarity is a big deal to understanding mixtures?

We reformulated "The chemical fingerprints approach used here, and other similar ones also based on structural similarity of the compounds [79], may be useful to suggest potential mechanisms of action of a compound, and it could aid in focusing on specific components of a mixture to reduce the experimental testing load that is tremendous." in the discussion (lines 469-472)

- Further discussion of how the study for Scandinavian population is going to help NAMs and how does this help with the wildlife population? LN 445 to LN 448 does not make sense.

Our study is obviously not specific to Scandinavians. We reformulated

"however it does have potential implications for regulatory agencies, both dealing both with environmental and human health regulations, that are moving towards non-testing studies, as laid out for example in the New Approach Methodologies [88] for human risk assessment." (lines 476-479)

"Our study helps to understand the potential effects caused by exposure to a realistic mixture of POPs, an exposure scenario that could be reached in particular populations (or subgroups within a population) of humans or wildlife, especially those living by the ocean and relying heavily on fish as a food source [89-92](line 483-486.
